# Therapeutic MK-4482/EIDD-2801 Blocks SARS-CoV-2 Transmission in Ferrets

**DOI:** 10.21203/rs.3.rs-89433/v1

**Published:** 2020-10-12

**Authors:** Robert M. Cox, Josef D. Wolf, Richard K. Plemper

**Affiliations:** 1 Institute for Biomedical Sciences, Georgia State University, Atlanta, GA

## Abstract

The COVID-19 pandemic is having a catastrophic impact on human health. Widespread community transmission has triggered stringent distancing measures with severe socioeconomic consequences. Gaining control of the pandemic will depend on interruption of transmission chains until protective herd immunity arises. Ferrets and related members of the weasel genus transmit SARS-CoV-2 efficiently with minimal clinical signs, resembling spread in the young-adult population. We previously reported an orally efficacious nucleoside analog inhibitor of influenza viruses, EIDD-2801 (or MK-4482), that was repurposed against SARS-CoV-2 and is in phase II/III clinical trials. Employing the ferret model, we demonstrate in this study high SARS-CoV-2 burden in nasal tissues and secretions that coincides with efficient direct-contact transmission. Therapeutic treatment of infected animals with twice-daily MK-4482/EIDD-2801 significantly reduced upper respiratory tract SARS-CoV-2 load and completely suppressed spread to untreated contact animals. This study identifies oral MK-4482/EIDD-2801 as a promising antiviral countermeasure to break SARS-CoV-2 community transmission chains.

The coronavirus disease (COVID)-19 pandemic is exerting a global impact on human health not experienced from a single pathogen since the Spanish flu outbreak of 1918. The etiologic agent, SARSCoV-2, has spread to over 35.5 million people to date, causing over 1 million deaths and substantial morbidity, and having an unprecedented catastrophic effect on societies and the global economy^1^. Interrupting widespread community transmission is paramount to establishing pandemic control and relaxing social-distancing measures. However, no vaccine prophylaxis is yet available and approved antiviral treatments such as remdesivir and reconvalescent serum cannot be delivered orally^2,3^, making them poorly suitable for transmission control.

We recently reported the development of MK-4482/EIDD-2801^4,5^, the orally available pro-drug of the nucleoside analog *N*^*4*^-hydroxycytidine (NHC), which has shown potent anti-influenza virus activity in mice, guinea pigs, ferrets, and human airway epithelium organoids^4,6,7^. Acting through induction of error catastrophe in virus replication^4,8^, NHC has broad-spectrum anti-RNA virus activity and is currently being tested in advanced clinical trials (NCT04405570 and NCT04405739) for the treatment of SARS-CoV-2 infection. In addition to ameliorating acute disease, we have demonstrated in a guinea pig transmission model that NHC effectively blocks influenza virus spread from infected animals to untreated contact animals^7^.

Several mouse models of SARS-CoV-2 infection have been developed, some of which were employed to confirm *in vivo* efficacy of MK-4482/EIDD-2801 also against beta-coronaviruses^9^. However, human SARS-CoV-2 cannot productively infect mice without extensive viral adaptation or introduction of human ACE2 into transgenic animals, and none of the mouse models supports transmission to uninfected mice^10^. Spillover of SARS-CoV-2 to farmed minks, subsequent large-scale mink-to-mink transmission and, in some cases, zoonotic transmission back to humans revealed efficient viral spread among members of the weasel genus without prior adaptation^11–14^. Although mink farms reported elevated animal mortality and gastrointestinal and respiratory clinical signs^15^, outbreak follow-up revealed continued intra-colony spread for extended periods of time^14^, suggesting that acute clinical signs in the majority of infected animals may be mild or absent. These mink field reports corroborated results obtained with experimentally infected ferrets showing that mustelids of the weasel genus transmit SARS-CoV-2 efficiently without strong clinical disease manifestation^16,17^. This presentation of SARS-CoV-2 infection resembles the experience of frequently asymptomatic or mildly symptomatic SARS-CoV-2 spread in the human young-adult population^18^.

In this study, we have explored the efficacy of oral MK-4482/EIDD-2801 against SARS-CoV-2 in the ferret model. We demonstrate significant reduction of upper respiratory tract virus load in animals treated therapeutically with MK-4482/EIDD-2801. Whereas SARS-CoV-2 efficiently spread to all contacts of vehicle-treated source animals, MK-4482/EIDD-2801 treatment blocked all SARS-CoV-2 transmission. These results support the administration of MK-4482/EIDD-2801 to asymptomatic or mildly symptomatic SARS-CoV-2 positives to rapidly block community transmission chains in addition to the treatment of patients with advanced clinical signs or severe disease.

## Efficient replication and shedding of SARS-CoV-2 in the ferret upper respiratory tract

**Fig. 1.**
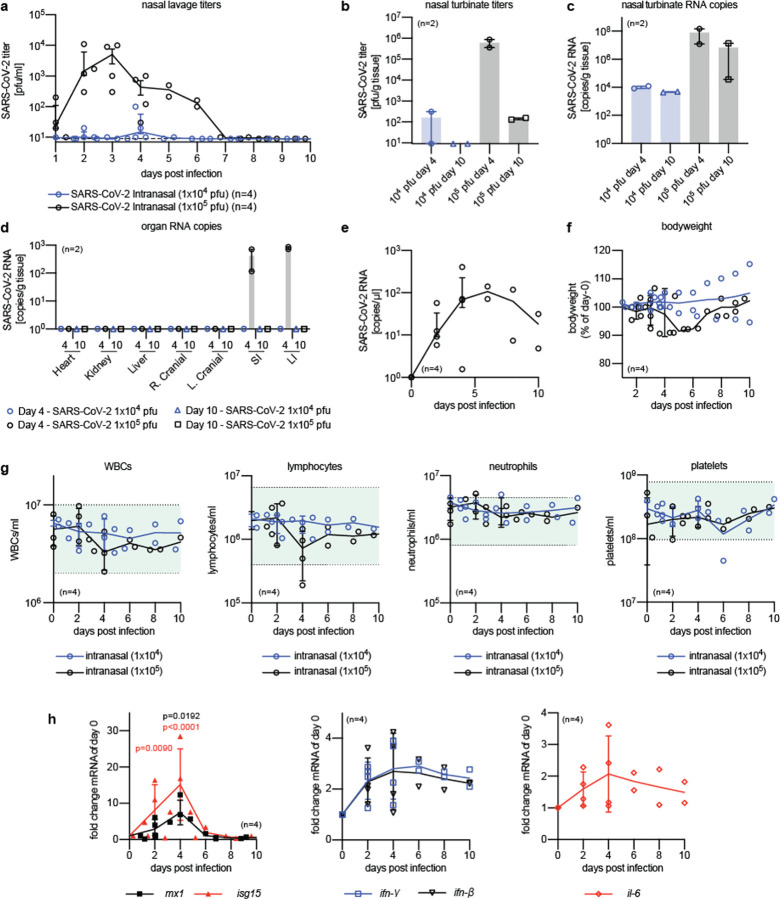
SARS-CoV-2 infects the upper respiratory tract of ferrets. Ferrets (n=4) were inoculated intranasally with 1×10^4^ or 1×10^5^ pfu of 2019-nCoV/USA-WA1/2020. **a**, Virus titer in nasal lavages collected daily. **b-f**, At 4 and 10 days post infection, 2 ferrets were sacrificed in each group and infection was characterized. **b**, Infectious virus particles in nasal turbinates. **c**, Viral RNA was present in the nasal turbinates of all infected ferrets. **d**, RT-qPCR quantitation of viral RNA copies in selected organs, two lung lobes (right (R.) and left (L.) cranial) per animal, and small (SI) and large (LI) intestine samples extracted from infected ferrets four or 10 days after infection. **e**, Detection of 2019-nCoV/USAWA1/2020 RNA in rectal swabs of ferrets inoculated with 1×10^5^ pfu. **f**, Bodyweight of ferrets, measured daily and expressed as % of weight at day 0. **g**, Complete blood count analysis, performed every second day. No noticeable differences were detected for all parameters tested, including total WBCs, lymphocytes, neutrophils, and platelets. The shaded green areas represent normal Vetscan HM5 lab values. **h**, Selected interferon and cytokine responses in PBMCs harvested every two days after infection. Analysis by qPCR for animals infected with 1×10^5^ pfu of 2019-nCoV/USA-WA1/2020. Infected ferrets displayed elevated expression of interferon stimulated genes (*mx1* and *isg15* (h; left)), *ifn-β* and *ifn-γ* (h; center), and *il-6* (h; right). Statistical analysis by two-way ANOVA with Dunnett’s post-hoc multiple comparison test. In all panels, symbols represent independent biological repeats (individual animals), lines connect group medians ± SEM (a,e) or SD (f-h), and bar graphs (b-d) show means ± range.

To validate host invasion and tissue tropism of SARS-CoV-2 in ferrets, we inoculated animals intranasally with 1×10^4^ or 1×10^5^ plaque-forming units (pfu) of SARS-CoV-2 clinical isolate 2019-nCoV/USA-WA1/2020 per animal. Shed virus burden was monitored daily over a 10-day period and virus load in the upper and lower respiratory tract determined on days four and ten after infection. In animals of the high inoculum group, virus release from the upper respiratory tract peaked three days after infection and was undetectable by day seven (Fig. 1a). No efficient infection was noted in the low inoculum group. Shedding profiles closely correlated with infectious particle load in nasal turbinates; a heavy virus tissue burden in the high inoculum group was present on day 4, which greatly decreased by approximately four orders of magnitude by day 10 (Fig. 1b).

**Extended Data Fig. 1.**
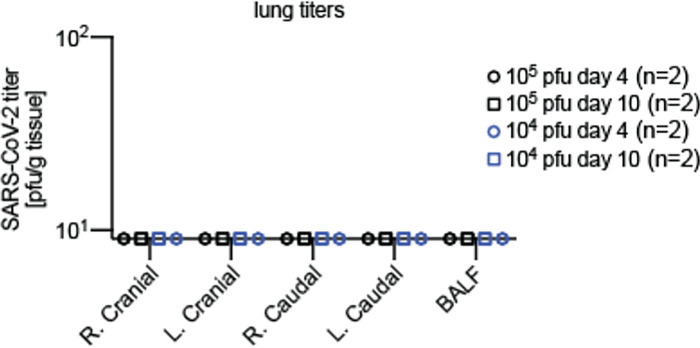
SARS-CoV-2 does not progress to the ferret lower respiratory tract. **a**, Analysis of bronchioalveolar lavages (BALF) and four lung lobes (right (R.) and left (L.) cranial and caudal) per ferret. BALF and tissues samples were harvested 4 (n=2) and 10 (n=2) days after infection. Symbols represent independent biological repeats (individual animals).

Low inoculum resulted in light virus load in the turbinates on day 4 and undetectable burden thereafter. However, qPCR-based quantitation of viral RNA copy numbers in the turbinates revealed continued presence of a moderate (approx. 10^4^ copies/g tissue) to high (≥10^7^ copies/g tissue) virus load after low and high inoculum, respectively (Fig. 1c). Independent of inoculum amount, no infectious particles were detected in bronchoalveolar lavages or lung tissue samples (extended data Fig. 1). At both days 4 and 10, several organ samples (lung, heart, kidney, liver) were also qPCR-negative (Fig. 1d), confirming inefficient infection of the ferret lower respiratory tract and limited systemic host invasion. Only small and large intestine samples were PCR-positive on day 4 after infection, and rectal swabs showed continued low-grade shedding of viral genetic material (Fig. 1e).

Animals in the high-inoculum group experienced a transient drop in body weight that reached a low plateau on days 5–6 after infection, but fully recovered by the end of study (Fig. 1f). No other clinical signs such as fever or respiratory discharge were noted. Complete blood counts taken every second day revealed no significant deterioration from the normal range in either inoculum group in overall white blood cells counts and lymphocyte, neutrophil, and platelet populations (Fig. 1g). Relative expression levels of type I and II interferon and IL-6 in ferret peripheral blood mononuclear cells (PBMCs) sampled in 48-hour intervals reached a plateau approximately 3 days after infection and stayed moderately elevated until the end of the study (Fig. 1h). Selected interferon-stimulated genes (ISGs) with antiviral effector function (MX1 and ISG15) showed a prominent expression peak four days after infection, followed by return to baseline expression by study end.

## Efficacy of MK-4482/EIDD-2801 against SARS-CoV-2 in ferrets

**Fig. 2.**
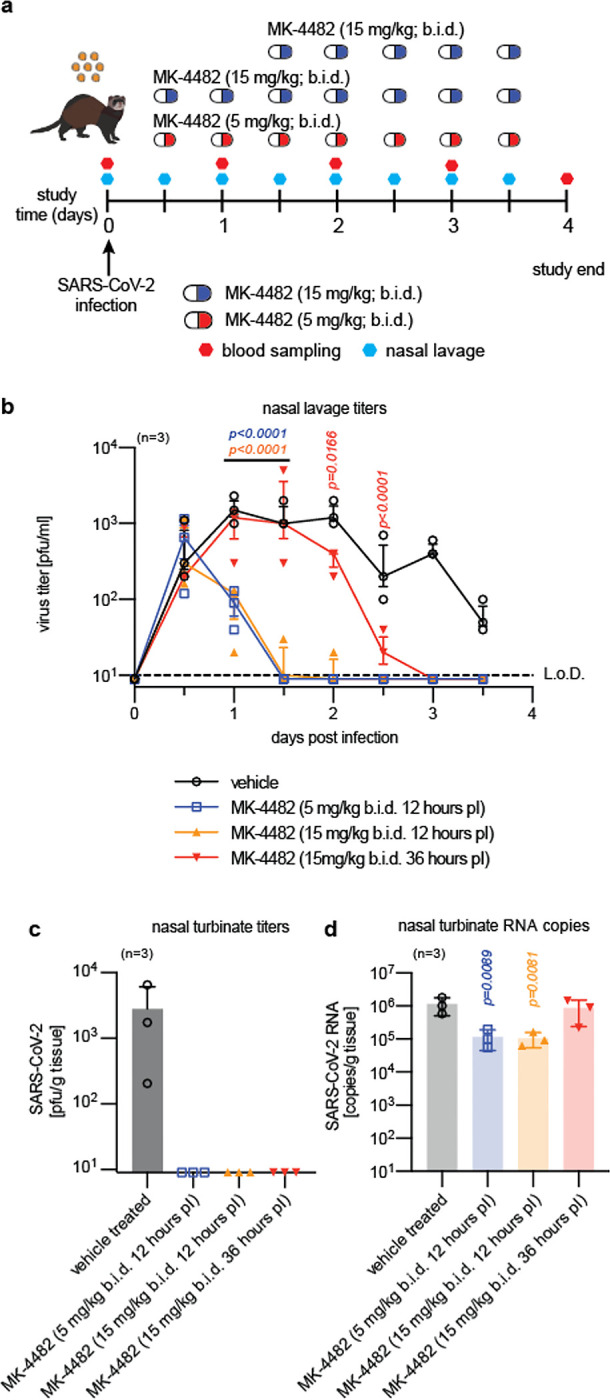
Therapeutic MK-4482/EIDD-2801 is orally efficacious against SARS-CoV-2 in ferrets. **a**, Therapeutic efficacy study schematic. Ferrets (n=3) were infected intranasally with 1×10^5^ pfu 2019-nCoV/USA-WA1/2020 and either gavaged with vehicle or treated *b*.*i*.*d*. with MK-4482/EIDD-2801 commencing 12 (5 mg/kg and 15 mg/kg) or 36-hours (15 mg/kg) after infection. Nasal lavages were collected twice daily. Blood was collected every other day. **b**, Viral nasal lavage titers in infected ferrets from (a). Treatment with MK-4482/EIDD-2801 significantly reduced virus titers within 12 hours dosing onset in all treatment groups. Statistical analysis by two-way ANOVA with Dunnett’s multiple comparison post-hoc test. P values are shown. **c-d**, Quantitation of infectious particles (c) and virus RNA copy numbers (d) in nasal turbinates of infected ferrets extracted four days after infection. Statistical analysis by one-way ANOVA with Dunnett’s multiple comparison post-hoc test. P values are shown. In all panels, symbols represent independent biological repeats (individual animals), lines connect group medians ± SEM (b), and bar graphs (c-d) show means ± SD.

Informed by these results, ferrets were infected in subsequent MK-4482/EIDD-2801 efficacy tests with 1×10^5^ pfu/animal and infectious virions in nasal lavages determined twice daily (Fig. 2a). Viral burden in respiratory tissues was assessed four days after infection. In all treatment experiments, MK-4482/EIDD-2801 was administered twice daily (*b*.*i*.*d*.) through oral gavage. Dosing commenced 12 hours after infection at 5 or 15 mg/kg body weight, or 36 hours after infection at 15 mg/kg. Shed viral titers in nasal lavages were equivalent in all MK-4482/EIDD-2801 groups and vehicle-treated controls at the time of first treatment start (12 hours after infection), indicating uniform inoculation of all animals in the study (Fig. 2b). Initiation of therapy at the 12-hour time point resulted in a significant reduction (p<0.001) of shed virus load within 12 hours, independent of the MK-4482/EIDD-2801 dose level administered, and infectious particles became undetectable within 24 hours of treatment start. When first administered at the peak of virus shedding (36 hours after infection), MK-4482/EIDD-2801 completely suppressed release of infectious virions into nasal lavages within a slightly longer 36-hour period, whereas vehicle control animals continued to shed infectious particles until study end.

**Extended Data Fig. 2.**
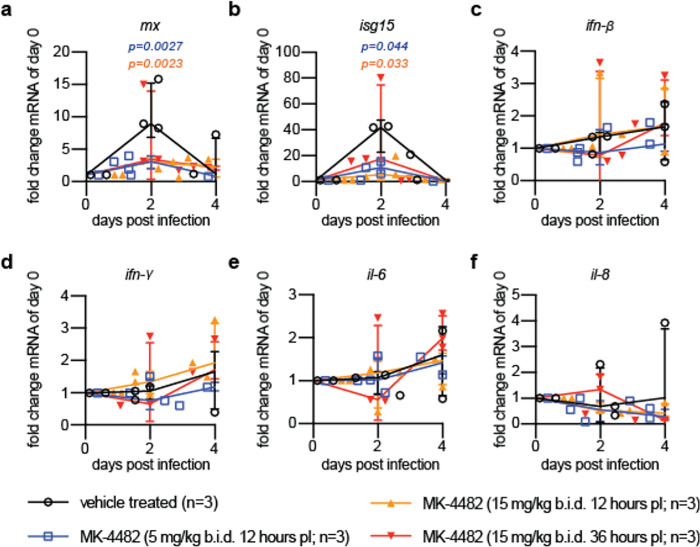
Interferon induction and cytokine profiling of SARS-CoV-2 ferrets treated with MK-4482/EIDD-2801. **a-f**, Selected interferon and cytokine expression levels in PBMCs relative to day 0. Blood samples of animals treated with MK-4482/EIDD-2801 or vehicle as specified were collected every two days after infection and PBMCs analyzed by RT-qPCR. Statistical analysis of changes relative to day 0 by two-way ANOVA with Dunnett’s post-hoc multiple comparison test. In all panels, symbols represent independent biological repeats (individual animals), lines connect group medians ± SD.

By 3.5 days after infection, only vehicle-treated animals carried detectable virus burden in nasal turbinates (Fig. 2c), indicating that MK-4482/EIDD-2801 had silenced all SARS-CoV-2 replication. SARS-CoV-2 RNA was still detectable in nasal tissues extracted from animals of all groups, albeit significantly reduced (p=0.0089 and p=0.0081 for the 5 mg/kg and 15 mg/kg MK-4482/EIDD-2801 groups, respectively) in treated animals versus the vehicle controls (Fig. 2d). Animals of the 12-hour therapeutic groups showed a significant reduction (p≤0.044) in effector ISG expression compared to vehicle-treated animals, although no significant differences in relative interferon and IL-6 induction were observed (extended data Fig. 2).

**Extended Data Fig. 3.**
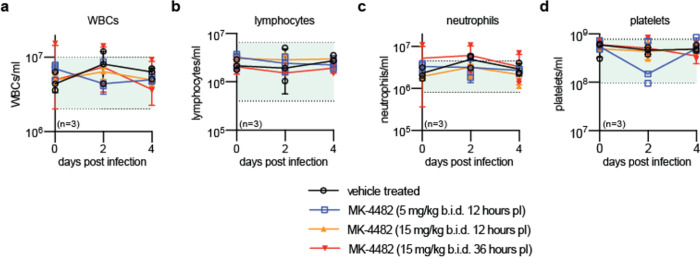
Complete blood count of SARS-CoV-2 ferrets treated with MK-4482/EIDD-2801. **a-d**, Blood samples were collected every two days after infection and complete blood counts determined. No abnormal values were observed in all parameters tested, including total WBCs (a), lymphocytes (b), neutrophils (c), and platelets (d). The shaded green areas represent normal Vetscan HM5 lab values. Symbols represent independent biological repeats (individual animals), lines connect group medians ± SD.

These results demonstrate oral efficacy of therapeutically administered MK-4482/EIDD-2801 against acute SARS-CoV-2 infection in the ferret model. Consistent with our previous pharmacokinetic (PK) and toxicology work-up of MK-4482/EIDD-2801 in ferrets, treatment did not cause any phenotypically overt adverse effects and white blood cell and platelet counts of drug-experienced animals remained in the normal range (extended data Fig. 3).

## Efficient direct contact transmission of SARS-CoV-2 between ferrets

**Fig. 3.**
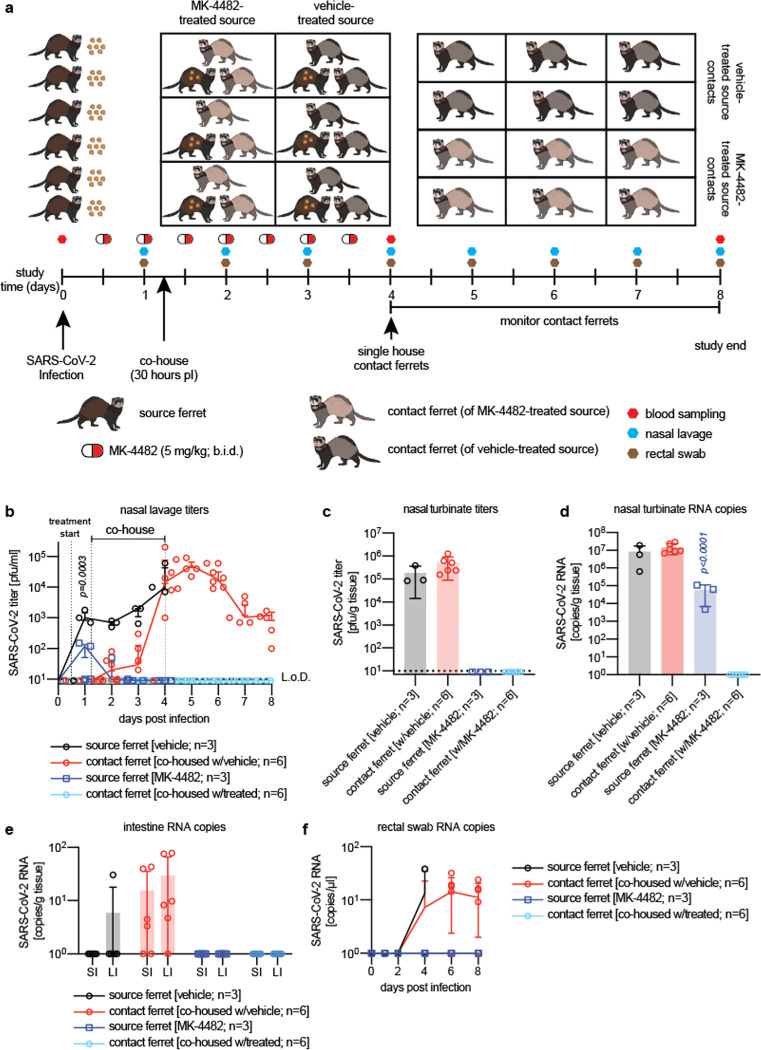
Therapeutic oral treatment with MK-4482/EIDD-2801 prevents contact transmission. **a**, Contact transmission study schematic. Two groups of source ferrets (n=3 each) were infected with 1×10^5^ pfu of 2019-nCoV/USA-WA1/2020 and received MK-4482/EIDD-2801 treatment (5 mg/kg *b*.*i*.*d*.) or vehicle starting 12 hours after infection. At 30 hours after infection, each source ferret was co-housed with two uninfected, untreated contact ferrets. After three days, source animals were euthanized and contact ferrets isolated and monitored for four days. Nasal lavages and rectal swabs were collected once daily and blood sampled at 0, 4, and 8 days post infection. **b**, Source ferrets treated with MK-4482/EIDD-2801 had significantly lower virus titers 12 hours after treatment onset (p=0.0003) than vehicle animals. Contacts of vehicle-treated sources began to shed 2019-nCoV/USA-WA1/2020 within 20 hours of co-housing. No virus was detectable in untreated contact of MK-4482/EIDD-2801-treated source ferrets. Statistical analysis by two-way ANOVA with Sidak’s multiple comparison post-hoc test. P values are shown. **c-d**, Quantitation of infectious particles (c) and virus RNA copy numbers (d) in nasal turbinates of source and contact ferrets from (b), extracted four and eight days after study start, respectively. Statistical analysis by one-way ANOVA with Sidak’s multiple comparison post-hoc test. **e-f**, Quantitation of virus RNA copy numbers in small (SI) and large (LI) intestines (e) and rectal swabs (f). Samples of MK-4482/EIDD-2801-treated source ferrets and their contacts were PCR-negative for viral RNA. In all panels, symbols represent independent biological repeats (individual animals), lines connect group medians ± SEM (b) or SD (f), and bar graphs (c-e) show means ± SD.

SARS-CoV-2 shedding into the ferret upper respiratory tract establishes conditions for productive spread from infected source to uninfected contact animals^16,17^. To assess transmission efficiency, we co-housed intranasally infected source animals with two uninfected contact animals each for a 3-day period, starting 30 hours after source animal inoculation (Fig. 3a). Nasal lavages and rectal swabs were obtained from all animals once daily and blood sampled at study start and on days four and eight after the original infection. Viral burden and RNA copy numbers in respiratory tissues were determined at the end of the co-housing phase (source animals) and at study end (contact animals).

Infectious particles first emerged in nasal lavages of some contact animals 24 hours after the start of co-housing (Fig. 3b). By the end of the co-housing phase, all contact animals were infected and approached peak virus replication phase, demonstrating that SARS-CoV-2 transmission among ferrets is rapid and highly efficient.

## MK-4482/EIDD-2801 prevents viral spread to untreated contact animals

A second cohort of source animals inoculated in parallel with SARS-CoV-2 received oral MK-4482/EIDD-2801 at the 5 mg/kg body weight dose level, administered *b*.*i*.*d*. starting 12 hours after infection. Productive infection of these animals was validated by SARS-CoV-2 titers in nasal lavages one day after infection (Fig. 3b) that very closely matched those seen in the initial efficacy tests (Fig. 2b). Although we also co-housed the treated source animals for nearly 3 days with two untreated contacts each, no infectious SARS-CoV-2 particles were detected in any of the series of nasal lavages obtained from these contacts or in any of the contact animal nasal turbinates sampled at study end (Fig. 3c).

Nasal turbinates extracted from the contacts of vehicle-treated source animals contained high viral RNA copy numbers, underscoring successful host invasion after transmission (Fig. 3d). Consistent with our earlier observations, turbinates of treated source animals harbored moderate to high (≥10^5^ copies/g tissue) amounts of viral RNA although infectious particles could not be detected. In contrast, all respiratory tissues of the contacts co-housed with MK-4482/EIDD-2801-treated source animals remained SARS-CoV-2 genome free, indicating the absence of any low-grade virus replication that could have hypothetically progressed in these animals below the detection level of infectious particles (Fig. 3e,f). Low SARS-CoV-2 RNA copy numbers were furthermore present in intestine tissue samples and rectal swabs of the vehicle source animals and their contacts, but were undetectable in the MK-4482/EIDD-2801-treated source group and co-housed contact animals.

## Discussion

Representatives of a number of animal species such as non-human primates^19^, dogs^20^, cats^20^, ferrets^20^, hamsters^21–23^, and bats^16^ were susceptible to SARS-CoV-2 without prior species adaptation when infected experimentally. Natural infection has been documented for felines^24^, dogs^25^ and minks^12,14^. Phylogenetic analysis of outbreaks in mink farms revealed prolonged intra-colony circulation and zoonotic mink-to-human transmission^14^, driving our selection of ferrets, members of the weasel genus closely related to minks, as a relevant SARS-CoV-2 transmission model.

We noted strong viral inoculum amount-dependence of experimental infection of ferrets. Productive host invasion characterized by robust virus replication in the upper respiratory tract and appearance of viral genetic material in gastrointestinal samples was only observed after intranasal delivery of 100,000 pfu of SARS-CoV-2. By comparison, natural infection through direct contact was far more efficient, to which prolonged exposure of contact to source animals may have been a contributing factor. However, nearly all contacts started to shed virus within less than 24 hours after the beginning of co-housing. This timeline indicates that transmission must have occurred in most cases immediately after introducing contact to source animals, despite the fact that shed viral titers of source animals were only 10^3^ pfu/ml nasal lavage in this disease period.

Independent of experimental versus natural infection, none of the SARS-CoV-2 infected ferrets displayed prominent clinical signs. The mink farm outbreaks may allow better appreciation of the clinical spectrum of SARS-CoV-2 in weasels, since data are based on a far greater number of animals. Whereas only a small subset of the thousands of infected minks displayed severe respiratory signs, most of those that died at the peak of farm outbreaks had developed acute interstitial pneumonia^12,15^. Possibly a consequence of mild disease in ferrets, our complete blood counts showed no robust lymphopenia, a prominent correlate of severe human SARS-CoV-2 disease^26,27^.

MK-4482/EIDD-2801 is currently being tested in advanced multi-center clinical trials (NCT04405570 and NCT04405739), which explore drug efficacy in lowering virus shedding in SARS-CoV-2-positive non-hospitalized and hospitalized patients, respectively. These studies were launched after successful completion of phase 1 safety trials (i.e. NCT04392219). Although dose levels applied in these studies and human PK data have not yet been disclosed, Merck & Co. have released^28^ that NHC blood levels were safely reached in humans that exceed antiviral concentrations against SARS-CoV-2 in primary human airway epithelia cultures (NHC EC_90_ approx. 0.5–1 μM^9^). Our PK profiles for MK-4482/EIDD-2801 revealed that NHC plasma concentrations ≥0.5 μM at trough (12 hours after dosing based on a *b*.*i*.*d*. regimen) are reached after oral dose levels of approximately 130 mg/kg and 10 mg/kg in cynomolgus macaques and ferrets, respectively^4^. These calculations drove our decision to dose ferrets at the 5 mg/kg level in this study, which represents a conservative estimate of a safe human dose equivalent based on all available information. By coincidence, 5 mg/kg is close to the lowest efficacious dose of MK-4482/EIDD-2801 against seasonal and pandemic influenza viruses in ferrets^4,6^, underscoring the high broad-spectrum antiviral potential of the drug.

Closely resembling our prior experience with influenza therapy^4,6^, MK-4482/EIDD-2801 was well tolerated and orally efficacious against SARS-CoV-2, reducing upper respiratory virus load below detection level within 24 hours of first drug administration when therapy was initiated after the onset of virus shedding, and by nearly two orders of magnitude when first administered at the peak of virus replication. Viral genetic material in gastrointestinal samples was likewise undetectable in treated animals, which is consistent with previous observations of sustained presence of the biologically active triphosphate form of NHC in all soft tissue but liver in different species^4,8,29^.

Importantly, treatment suppressed all transmission to untreated direct contacts, despite prolonged direct proximity of source and contact animals and detectable virus shedding from source animals at the beginning of the co-housing phase. This complete transmission block may indicate a bottom threshold of shed SARS-CoV-2 load for successful spread. Since the antiviral effect of NHC arises from induction of error catastrophe^4,7,8^, it is also possible that genome integrity of some EIDD-2801-experienced virions shed from treated animals was only partially compromised. Incorporated NHC base pairs as cytosine or uracil due to spontaneous tautomeric interconversions^30^. Limited presence of the analog in viral genomes generated shortly after treatment start could have still allowed virus replication on cultured cells for titration, but not successful host invasion.

Our prior studies with influenza viruses demonstrate that the MK-4482/EIDD-2801-mediated block of respiratory viral transmission is not host species-restricted. Oral treatment with MK-4482/EIDD-2801 or NHC reduced shed influenza virus titers in ferret nasal lavages with potency and kinetics comparable to the effect seen here against SARS-CoV-2^4^ and effectively prevented influenza virus direct contact transmission between guinea pigs^7^. If ferret-based inhibition of SARS-CoV-2 transmission by MK-4482/EIDD-2801 is predictive of the antiviral effect in humans, COVID-19 patients could become non-infectious within 24 to 36 hours after the onset of oral treatment. In addition to the direct therapeutic promise of alleviating clinical disease, a shortened shedding period would safely allow reduction of isolation times of SARS-CoV-2 positives and narrow the window of opportunity for viral transmission. Treatment with MK-4482/EIDD-2801, in particular when initiated early after infection, thus has the potential to provide three-fold benefit: it may mitigate the risk of progression to severe disease and accelerate recovery, ease the emotional and socioeconomic toll associated with mandatory prolonged isolation, and aid in rapidly silencing local outbreaks.

## Methods

### Study design

Ferrets were used as an *in vivo* model to examine efficacy of therapeutically administered oral MK-4482/EIDD-2801 against SARS-CoV-2 infection and virus transmission to uninfected contact animals. Viruses were administered to source animals through intranasal inoculation and virus load monitored periodically in nasal lavages and rectal swabs, and 4 or 10 days after exposure in respiratory tissues and a subset of organs. Virus titers were determined based on plaque assay and viral RNA copy numbers, blood samples subjected to CBC analysis and RT-qPCR quantitation of selected cytokine and innate antiviral effector expression levels.

### Cells and viruses

Vero-E6 cells were cultured in Dulbecco’s modified Eagle’s medium (DMEM) supplemented with 7.5% heat inactivated fetal bovine serum (FBS) at 37°C with 5% CO_2_. SARS-CoV-2 (SARS-CoV-2/human/USA-WA1/2020) was propagated using Vero-E6 cells supplemented with 2% FBS. Virus stocks were stored at −80°C and titers were determined by plaque assay. Vero-E6 cells were routinely checked in 6-month intervals for bacterial and mycoplasma contamination.

### Plaque assay

Samples were serially diluted (10-fold starting at 1:10 initial dilution) in DMEM supplemented with 2% FBS containing antibiotics-antimycotics (Gibco). Serial dilutions were added to Vero-E6 cells seeded in 12-well plates at 3×10^5^ cells per well 24-hours prior. Virus was allowed to adsorb for 1 hour at 37°C. Subsequently, inoculum was removed, and cells were overlaid with 1.2% Avicel (FMC biopolymer) in DMEM and incubated for three days at 37°C with 5% CO_2_. Avicel was removed and cells were washed once with PBS, fixed with 10% neutral buffered formalin, and plaques were visualized using 1% crystal violet.

### Establishing infectious dose

Female ferrets (6–10 months of age) were purchased from Triple F Farms. Upon arrival, ferrets were rested for one week, then randomly assigned to groups and housed individually in ventilated negative pressure cages in an ABSL-3 facility. In order to establish a suitable inoculum for efficacy and transmission studies, ferrets (n=4) were inoculated intranasally with 1×10^4^ and 1×10^5^ pfu of 2019-nCoV/USA-WA1/2020 in 1 ml (0.5 ml per nare). Prior to inoculation, ferrets were anesthetized with dexmedetomidine/ketamine. Nasal lavages were performed once daily using 1 ml of PBS containing 2× antibiotics-antimycotics (Gibco). For blood sampling, ferrets were anesthetized with dexmedetomidine and approximately 0.5 ml blood was drawn from the anterior vena cava. Complete blood counts (CBC) were performed using a Vetscan HM5 (Abaxis) in accordance with the manufacturer’s protocol. Rectal swabs were performed every two days. Groups of two ferrets were sacrificed 4- and 10-days post infection and organs were harvested to determine virus titer and the presence of viral RNA in different tissues.

### *In vivo* efficacy of MK-4482/EIDD-2801 in ferrets

Groups of ferrets (n=3 each) were inoculated with 1×10^5^ pfu of 2019-nCoV/USA-WA1/2020 in 1 ml (0.5 ml per nare). At 12 hours after infection, three groups of ferrets were treated *b*.*i*.*d*. with vehicle (1% methylcellulose) or MK-4482/EIDD-2801 at a dose level of 5 mg/kg or 15 mg/kg, respectively. At 36 hours after infection, a fourth group of ferrets began receiving *b*.*i*.*d*. treatment with MK-4482/EIDD-2801 at a dose of 15 mg/kg. Compound was administered via oral gavage in 1% methylcellulose. After treatment onset, *b*.*i*.*d*. dosing was continued until four days after infection. Nasal lavages were performed on all ferrets every 12 hours. Blood samples were obtained every two days after infection and stored in K_2_-EDTA tubes (Sarstedt CB 300). CBC analysis was performed on each blood sample in accordance with the manufacturer’s protocols. After CBC analysis, red blood cells were lysed with ACK buffer (150 mM NH_4_CL, 10mM KHCO_3_, 0.01 mM EDTA pH 7.4) and PBMCs were harvested and stored at −80°C in RNAlater until further qPCR analysis was performed. Four days after infection, all ferrets were euthanized and organs harvested to determine virus titers and the presence of viral RNA in different tissues.

### Contact transmission of SARS-CoV-2 in ferrets

A group of 6 individually housed source ferrets were inoculated intranasally with 1×10^5^ pfu of 2019-nCoV/USA-WA1/2020. Twelve hours after infection, source ferrets were split into two groups (n=3 each) receiving vehicle or MK-4482/EIDD-2801 treatment at a dose of 5 mg/kg *b*.*i*.*d*. daily by oral gavage. At 30 hours post infection, each source ferret was co-housed with two uninfected and untreated contact ferrets. Ferrets were co-housed until 96 hours after infection, when source ferrets were euthanized and contact animals housed individually. Contact animals were monitored for four days after separation from source ferrets, then sacrificed. Nasal lavages and rectal swabs were performed every 24 hours on all ferrets. Blood samples were collected at 0, 4, and 8 days after source ferret infection. For all ferrets, organs were harvested to determine virus titers and the presence of viral RNA in different tissues.

### Titration of SARS-CoV-2 in tissue extracts

For virus titration, organs were weighed and homogenized in PBS. Homogenates were centrifuged for 5 minutes at 2,000×g at 4°C. Clarified supernatants were harvested and used in subsequent plaque assays. For detection of viral RNA, harvested organs were stored in RNAlater at −80°C. Tissues were ground and total RNA was extracted using a RNeasy mini kit (Qiagen). RNA was extracted from rectal swabs using the ZR Viral RNA Kit (Zymo Research) in accordance with the manufacturer’s protocols.

### SARS-CoV-2 RNA copy numbers

Detection of SARS-CoV-2 RNA was performed using the nCoV_IP2 primer-probe set (National Reference Center for Respiratory Viruses, Institut Pasteur, Paris) targeting the SARS-CoV-2 RdRp gene. An Applied Biosystems 7500 Real-Time PCR System using the StepOnePlus Real-Time PCR System was used to perform qPCR reactions. TaqMan Fast Virus 1-Step Master Mix (Thermo Fisher Scientific) was used in combination with the nCoV_IP2 primer-probe set to detect viral RNA. To quantitate RNA copy numbers, a standard curve was created using a PCR fragment (nucleotides 12669–14146 of the SARS-CoV-2 genome) generated from viral cDNA using nCoV_IP2 forward primer and the nCoV_IP4 reverse primer. RNA values were normalized based on weights of tissues used.

### Systemic interferon and cytokine profiling

Relative expression of interferon, interferon stimulated genes and cytokines was determined by real-time PCR analyses. RNA was extracted from PBMCs harvested at various after infection. cDNA was reverse transcribed with SuperScript III (Invitrogen) using oligo-dT primers and analyzed by real-time PCR using Fast SYBR Green Master Mix (Applied Biosystems). Signals were normalized to glyceraldehyde-3-phosphate dehydrogenase mRNA, analyzed by the comparative threshold cycle (ΔΔCt) method, and expressed relative to day 0 of infection for each respective animal. Sequences of the primers used for the analyses are shown in supplementary table S1.

### Statistical analysis

When comparing more than two groups, one-way analysis of variance (ANOVA) or two-way ANOVA with Dunnett’s or Sidak’s multiple comparison post hoc tests as specified in figure legends were used to assess statistical difference between samples. All statistical analyses were carried out in Prism version 8.4.3 (GraphPad). The number of individual biological replicates (n values) is shown in the figures and specified in the figure legends for each experiment. Representations of mean or median ± standard deviation are specified in the figure legends. The significance threshold (α) was set to 0.05. Exact P values are provided in the figures.

### Ethical compliance

All animal work was performed in compliance with the *Guide for the Care and Use of Laboratory Animals* of the National Institutes of Health and the Animal Welfare Act Code of Federal Regulations. Experiments with SARS-CoV-2 involving ferrets were approved by the Georgia State Institutional Animal Care and Use Committee under protocol A20031. All experiments using infectious SARS-CoV-2 were approved by the Georgia State Institutional Biosafety Committee under protocol B20016 and performed in a BSL-3/ABSL-3 facilities at Georgia State University.

## Extended Data

## Supplementary Material

Supplement
