# Supplementary material for: Therapeutic MK-4482/EIDD-2801 Blocks SARS-CoV-2 Transmission in Ferrets
Source: Res Sq. 2020 Oct 12:rs.3.rs-89433. Preprint. [Version 1] doi: 10.21203/rs.3.rs-89433/v1 (PMC7553152; doi:10.21203/rs.3.rs-89433/v1)
Supplement: Supplement [file 4ffd5b7365903c367a032fed.pdf]

Therapeutic MK-4482/EIDD-2801 Blocks SARS-CoV-2 Transmission in Ferrets

Robert M. Cox[1], Josef D. Wolf[1], Richard K. Plemper[1]*

[1]Institute for Biomedical Sciences, Georgia State University, Atlanta, GA

Supplementary Information

Supplementary Table 1. Primers used for qPCR.

Source Data File 1. Source data and statistical analyses of results shown in figures 1-3 and extended data figures 1-3.

Supplementary table 1. Primers used for qPCR of ferret samples.

primer ID	sequence
nCoV_IP2-12669_Fw	5'-ATGAGCTTAGTCCTGTTG-3'
nCoV_IP2-12759_Rv	5'-CTCCCTTTGTTGTGTTGT-3'
nCoV_IP2-12696 probe	[5']Fam-AGATGTCTTGTGCTGCCGGTA-[3']BHQ-1
nCoV_IP4-14146_Rv	5'-CTGGTCAAGGTTAATATAGG-3'
Ferret GAPDH_Fw	5'-AACATCATCCCTGCTTCCACTGGT-3'
Ferret GAPDH_Rv	5'-TGTTGAAGTCGCAGGAGACAACCT-3'
Ferret IL-6_Fw	5'-AGTGGCTGAAACACGTAACAATTC-3'
Ferret IL-6_Rv	5'-ATGGCCCTCAGGCTGAACT-3'
Ferret IL-8_Fw	5'-TGCTTTCTGCAGTTCTGTGTGAGC-3'
Ferret IL-8_Rv	5'-ATGTGGGCCACTGTCAATCACTCT-3'
Ferret ISG15_Fw	5'-AGCAGCAGATAGCCCTGAAA-3'
Ferret ISG15_Rv	5'-CAGTTCTTCACCACCAGCAG-3'
Ferret IFN-β_Fw	5'-GGTGTATCCTCCAAACTGCTCTCC-3'
Ferret IFN-β_Rv	5'-CACTCCACACTGCTGCTGCTTAG-3'
Ferret IFN-γ_Fw	5'-TCAAAGTGATGAATGATCTCTCACC-3'
Ferret IFN-γ_Rv	5'-GCCGGGAAACACACTGTGAC-3'
Ferret mx1_Fw	5'-ACATCCTCAGGCAGGAGACA-3'
Ferret mx1_Rv	5'-CAGGTCAGGCTTTGTCAAGA-3'
